# Pancreatic ductal adenocarcinoma with inferior vena cava invasion: a report of three resected cases

**DOI:** 10.1186/s40792-017-0348-5

**Published:** 2017-05-23

**Authors:** Takuya Mizumoto, Tadahiro Goto, Hirochika Toyama, Keitaro Sofue, Sadaki Asari, Sachio Terai, Motofumi Tanaka, Masahiro Kido, Tetsuo Ajiki, Takumi Fukumoto, Yonson Ku

**Affiliations:** Division of Hepato-Biliary-Pancreatic Surgery, Department of Surgery, Kobe University Graduate School of Medicine, 7-5-2, Kusunoki-cho, Chuo-ku, Kobe, 650-0017 Hyogo Japan

**Keywords:** Pancreatic ductal adenocarcinoma, Inferior vena cava, Invasion, Resection

## Abstract

**Background:**

Pancreatic ductal adenocarcinoma (PDAC) often infiltrates to the adjacent major vasculatures; however, direct invasion of PDAC to the inferior vena cava (IVC) is uncommon.

**Case presentation:**

We report our experience with three cases of PDAC directly invading the IVC wall. All three patients underwent pancreatoduodenectomy along with wedge resection of the IVC wall without severe postoperative complications. Histopathological studies revealed tumor infiltration to the adventitia of the IVC. All patients achieved negative surgical margins. One patient was still alive 26 months after surgery without tumor recurrence. Two patients experienced recurrence; one patient experienced liver metastasis but was still alive and in a stable condition without further tumor progression 12 months after surgery. Another patient experienced multiple liver metastasis 10 months after surgery and died 26 months after surgery.

**Conclusions:**

Pancreatoduodenectomy along with wedge resection of the IVC wall for patients with PDAC directly invading the adventitia of the IVC can be performed safely. Further accumulation of cases is needed to elucidate the prognostic impact of IVC invasion.

## Background

Pancreatic ductal adenocarcinoma (PDAC) has the worst prognosis among all gastrointestinal cancers. Surgical resection is the only possibly curative therapy; however, only 15 to 20% of pancreatic cancer is indicated for surgery with curative intent [1, 2]. This is due to not only distant metastasis but also local invasion to adjacent organs. PDAC frequently infiltrates the major vasculatures that exist posterior to the pancreas, such as the superior mesenteric artery, portal vein (PV), superior mesenteric vein (SMV), or common hepatic artery. These findings are considered significant in regulating the resectability of the tumors [3, 4]. However, direct invasion of the inferior vena cava (IVC) is uncommon. According to the National Comprehensive Cancer Network (NCCN) Clinical Practice Guidelines, tumor contact with the IVC is defined as borderline resectable; however, an unresectable status is not defined in accordance with IVC involvement and IVC invasion is not contraindicated for surgery [5]. Given the lack of reported cases, the radiological features, surgical implications, and oncological impact of PDAC which invade the IVC are unclear.

## Case presentation

### Case 1

**Fig. 1:**
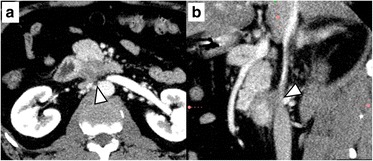
Preoperative computed tomography image of case 1. **a** Dense soft tissue of the pancreatic tumor contacting the ventral surface of the inferior vena cava (IVC) (*arrowhead*). **b** Sagittal multi-planar reformation showed that the interface between the tumor and the IVC was not well defined

**Fig. 2:**
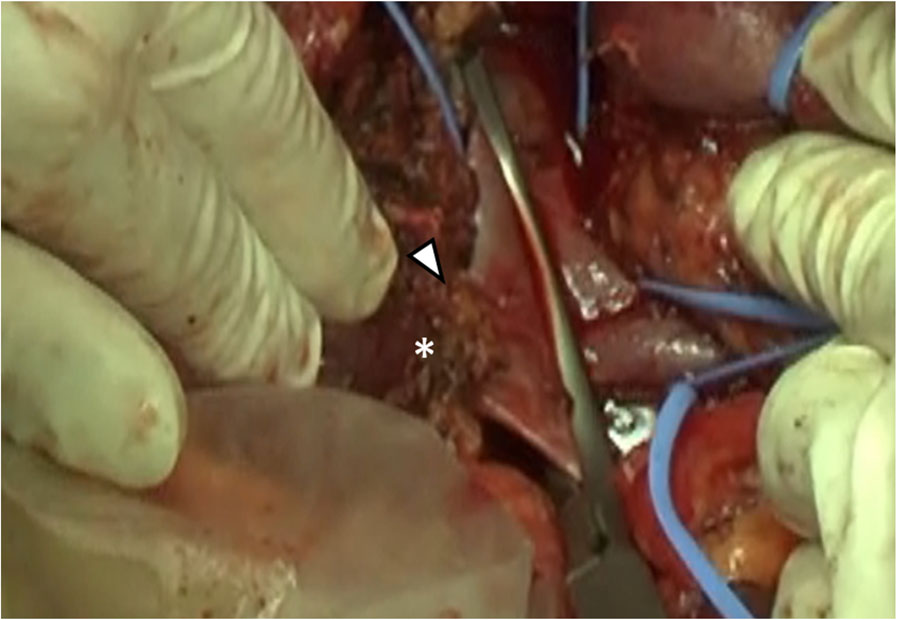
Intraoperative findings of case 1. Stiff attachment between the tumor (*asterisk*) and the inferior vena cava (IVC) was identified (*arrowhead*)

**Fig. 3:**
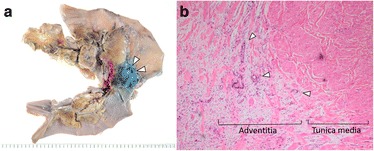
Macroscopic and microscopic findings of resected specimen of case 1. **a** Inferior vena cava (IVC) wall was resected together with the pancreatic head (*arrowhead*). **b** Tumor invasion of the adventitia of the IVC wall was observed (*arrowhead*) (hematoxylin and eosin stain, ×40)

**Fig. 4:**
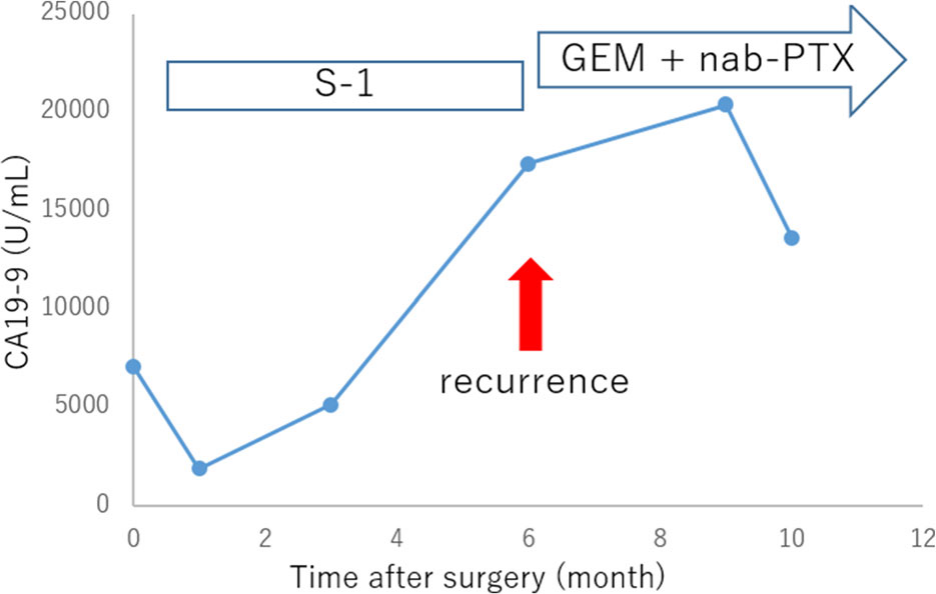
Changes in serum carbohydrate antigen 19–9 (CA19-9) level associated with postoperative chemotherapy in case 1. CA19-9 level of postoperative month 0 shown in the *graph* indicates the preoperative CA19-9 level

A 50-year-old man presented at our hospital with upper abdominal pain and loss of appetite. Computed tomography (CT) imaging demonstrated a hypovascular mass that measured 10 mm in diameter in the uncinate process of the pancreas. The dense soft tissue of the pancreatic lesion was in contact with the ventral surface of the IVC; however, signs of obvious invasion were not detected (Fig. 1). A self-expanding metallic stent was placed in the duodenum because the horizontal portion of the duodenum was obstructed by the tumor. Therefore, pancreatoduodenectomy (PD) was performed. During surgery, stiff attachment between the tumor and the IVC was identified and wedge resection of the IVC wall was performed via side clamping of the IVC (Fig. 2). Pathological studies of the surgical specimen revealed direct invasion by the PDAC to the adventitia of the IVC (Fig. 3). He was discharged without significant postoperative complications, including IVC thrombosis or leg edema, on postoperative day (POD) 25. He underwent adjuvant chemotherapy (S-1; 120 mg/day) for 4 months; however, the CT imaging performed 6 months after surgery identified liver metastasis that was treated with systemic chemotherapy (Gemcitabine, 1000 mg/m^2^; nab-Paclitaxel, 125 mg/m^2^). He was still alive and in stable condition without further tumor progression 12 months after surgery (Fig. 4).

### Case 2

**Fig. 5:**
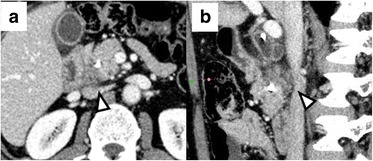
Preoperative computed tomography (CT) imaging of case 2. **a** An irregular tumor was identified in the pancreatic head and extended to the dorsal border of the pancreas (*arrowhead*). **b** Sagittal multi-planar reformation showed that the lesion adhered to and deformed the inferior vena cava (IVC)

**Fig. 6:**
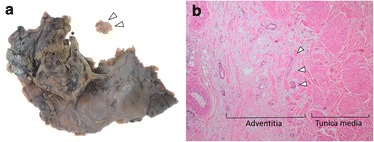
Macroscopic and microscopic findings of the resected specimen of case 2. **a** The inferior vena cava (IVC) wall was resected together with the pancreatic head and separated through the pathological examination (*arrowhead*). *Asterisk* shows the initial site of the resected IVC wall. **b** Tumor invasion of the adventitia of the IVC wall was observed (*arrowhead*) (hematoxylin and eosin stain, ×40)

**Fig. 7:**
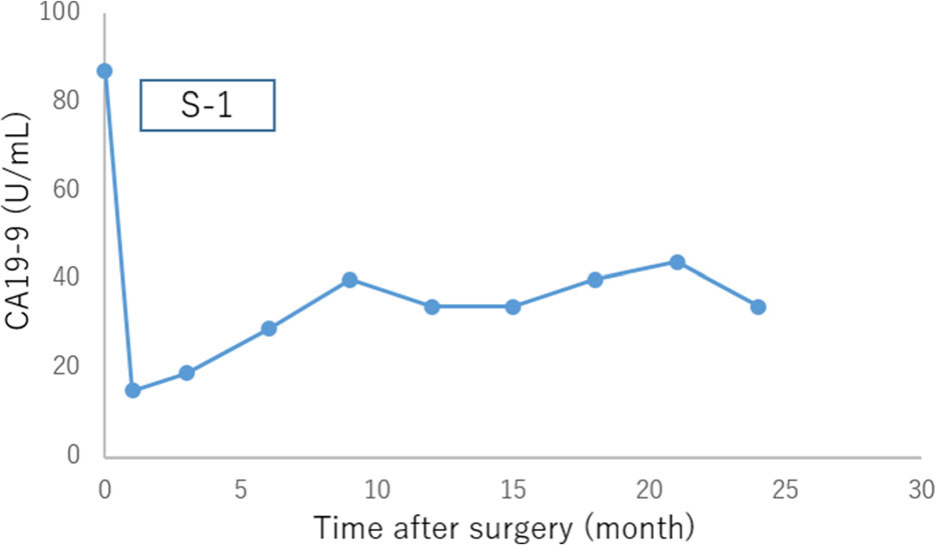
Changes in serum carbohydrate antigen 19–9 (CA19-9) level associated with postoperative chemotherapy in case 2. CA19-9 level of postoperative month 0 shown in the *graph* indicates the preoperative CA19-9 level

A 59-year-old man with obstructive jaundice was admitted to a different hospital. He was referred to our institution because of possible periampullary malignancies. CT imaging identified an irregular tumor (31 mm in diameter) in the pancreatic head that extended to the dorsal border of the pancreas (Fig. 5). The tumor was stiffly attached to the IVC wall; therefore, wedge resection of the IVC wall was performed via side clamping of the IVC. Pathological studies of the surgical specimen revealed PDAC directly invading the adventitia of the IVC (Fig. 6). He experienced postoperative pancreatic fistula (International Study Group of Postoperative Pancreatic Fistula grade B) that improved with conservative therapy and was discharged on POD 29 [6]. Complications related to IVC resection did not occur during follow-up. He underwent adjuvant systemic chemotherapy (S-1; 120 mg/day) for 6 months and was still alive without tumor recurrence 26 months after surgery (Fig. 7).

### Case 3

**Fig. 8:**
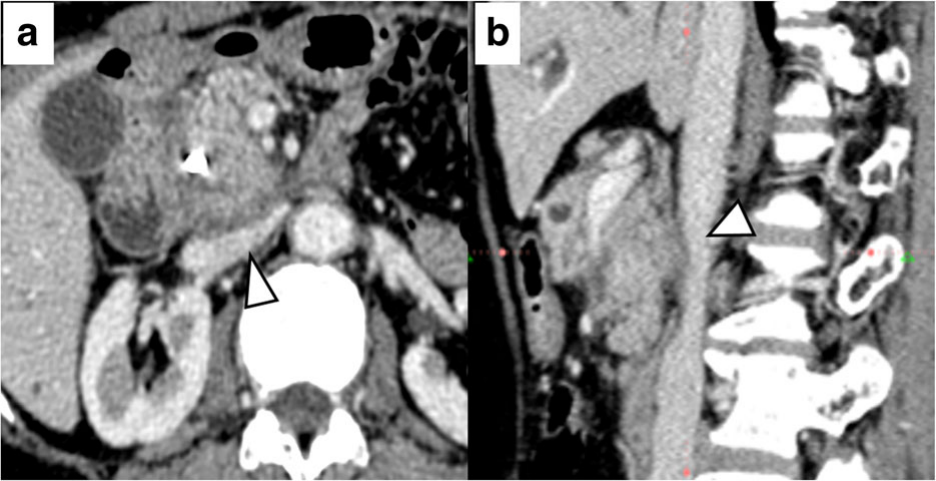
Preoperative computed tomography (CT) imaging of case 3. **a** Dense soft tissue connected to the pancreatic lesion widely contacting the ventral surface of the inferior vena cava (IVC) (*arrowhead*). **b**. Sagittal multi-planar reformation showed that the lesion adhered to and deformed the IVC

**Fig. 9:**
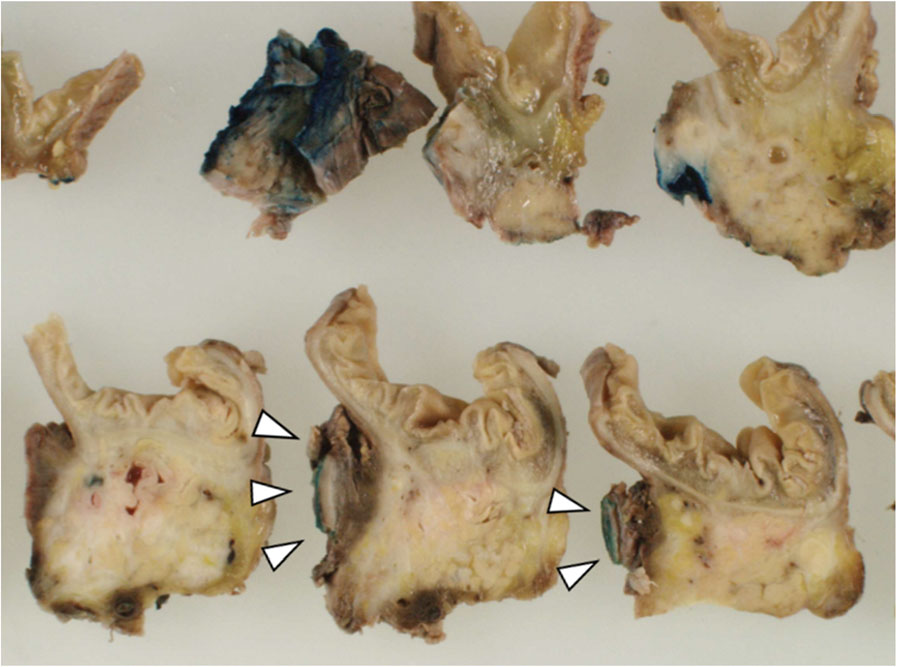
Macroscopic findings of resected specimen of case 2. The inferior vena cava (IVC) wall was resected together with the pancreatic head (*arrowhead*)

**Fig. 10:**
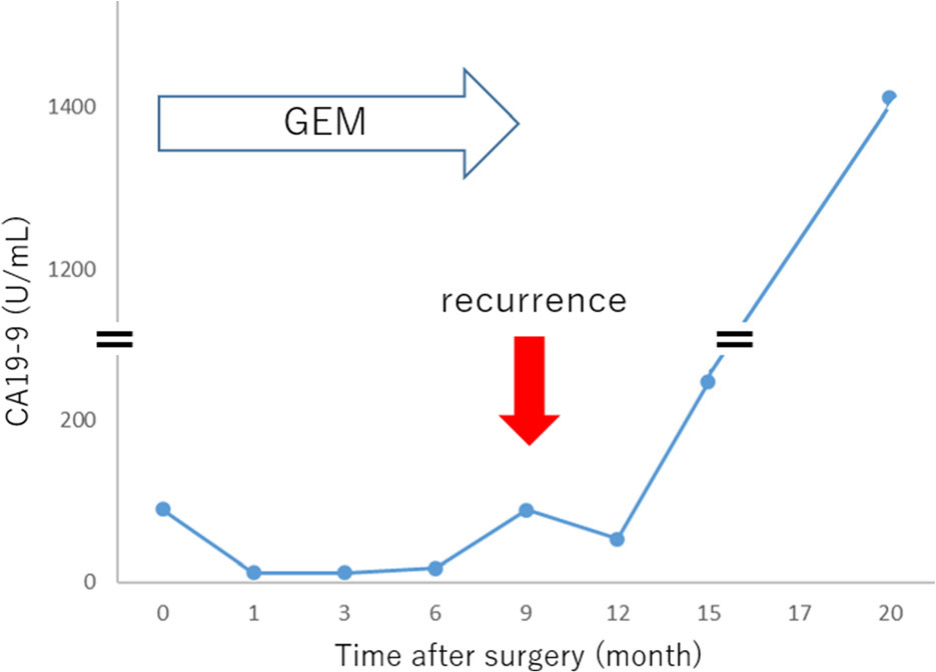
Changes in serum carbohydrate antigen 19–9 (CA19-9) level associated with postoperative chemotherapy in case 2. CA19-9 level of postoperative month 0 shown in the *graph* indicates the preoperative CA19-9 level

A 68-year-old woman with epigastric pain and jaundice presented to a different hospital but was later referred to our institution. CT imaging showed a hypovascular tumor (29 mm in diameter) in the pancreatic head (Fig. 8). The tumor widely contacted the IVC wall; however, there were no signs of obvious invasion. PDAC was suspected and she underwent PD. Intraoperative findings revealed a stiff attachment between the tumor and the IVC. Wedge resection of the IVC wall was performed using side clamping. In addition, combined resection and reconstruction of the SMV was performed because of suspicion for SMV invasion. Pathological findings of the surgical specimen revealed tumor infiltration of the adventitia of the IVC (Fig. 9). Surgical margins including IVC resection end were negative; however, tumor metastases to the para-aortic lymph nodes were identified. The postoperative course was uneventful, and she underwent systemic chemotherapy (Gemcitabine, 1000 mg/m^2^) beginning on POD 34. She experienced multiple liver metastases, bone metastasis 10 months after surgery, and died 16 months after recurrence (Fig. 10).

### Discussion

PDAC often infiltrates the adjacent major vasculatures; however, direct invasion of the IVC is rare. We experienced 3 cases of PDAC directly invading the IVC wall among a total of 212 cases of pancreatic resection for PDAC presented during the same period (1.4%). This observation is consistent with a previous study by Kitagawa and colleagues that reported 2 of 140 resected cases of PDAC (1.4%) with IVC invasion during pathologic studies [7]. Clinical features of these cases are presented in Table 1.

**Table 1:** Clinical features of three cases of PDAC invading the IVC

Case	Age, years	Sex	Preoperative CA19-9, U/ml	Operative procedures	Operation time	Bleeding, g	Tumor size, cm	Lymph node metastasis	Tumor differentiation	OS, months	Alive/dead
1	50	M	7075	PD + IVCR	8 h, 26 min	225	4.8	Positive	Well to moderate	12	Alive
2	59	M	87	PD + IVCR	9 h, 45 min	690	3.1	Positive	Moderate	26	Alive
3	68	F	90	PD + IVCR + SMVR	12 h, 32 min	2300	4.5	Positive	Moderate	26	Dead

*PDAC* pancreatic ductal adenocarcinoma, *IVC* inferior vena cava, *M* male, *F* female, *CA19-9* carcinoembryonic antigen 19–9, *PD* pancreatoduodenectomy, *IVCR* inferior vena cava resection, *SMVR* superior mesenteric vein resection, *OS* overall survival

The efficacy of concomitant IVC resection for patients with PDAC is unclear. Among the 3 cases reported in this study, surgical procedures were performed safely and no patient experienced complications related to IVC resection. Regarding oncological aspects, all three patients achieved negative surgical margins. Although one patient was identified to have para-aortic lymph node metastasis on pathological study, postoperative survival of our cases compared favorably with those of patients with other borderline resectable cases; the reported overall survival time after surgery for those with borderline resectable PDAC is almost 20 months [8, 9]. Moriura et al. reported two cases of PDAC invading IVC wall during IVC resection for hepato-biliary-pancreatic malignancies [10]. The reported prognosis of these two cases was dismal (death due to tumor recurrence 5 and 8 months after surgery, respectively); however, these cases were advanced tumors which needed total pancreatectomy or multivisceral resection and were difficult to simply compare with our cases. Nah et al. also reported a case of PDAC invading IVC wall; however, follow-up period of 8 months was not enough to evaluate postoperative prognosis [11]. Further, more such cases need to be reported in order to elucidate the prognostic impact of IVC invasion.

Kocher’s maneuver is usually performed during PD; surgeons dissect between the pancreatic head and IVC. Kitagawa et al. reported that only 3% of the PDAC directly infiltrates beyond the fusion fascia, which covers the IVC [7]. However, when tumors directly invade the IVC wall, dissecting between the IVC and pancreatic head may cause tumor remnants or seeding due to cutting into the tumor. Because of the desmoplastic nature of PDAC, it is difficult to distinguish fibrous adhesions from tumor involvement intraoperatively. A preoperative predictor of IVC invasion is required; however, there are no criteria that can effectively predict IVC invasion with preoperative imaging studies. According to the NCCN guidelines, PDAC in contact with the IVC is defined as borderline resectable. This definition may lead to a false-positive diagnosis of IVC invasion; PDAC in the pancreatic head occasionally contacts the IVC wall even when IVC invasion is negative. When retrospectively reviewed, the dense soft tissue of the infiltrating tumors widely contacted the IVC wall, and the IVC was deformed on sagittal imaging (Figs. 1b, 3b, and 4b). In cases of tumors contacting the IVC, evaluating the sagittal sections of CT images may help in the diagnosis of IVC invasion preoperatively.

In the case of PDAC largely invading the IVC on preoperative imaging, the indication for surgery should be determined more carefully. In the literature, severe complications such as massive bleeding, gas embolism, venous stenosis, and thrombosis are predominantly associated with segmental resection of the IVC in the setting of liver resection [12, 13]. Additionally, the oncologic significance of massive IVC invasion is unknown. There can be a potential risk of early pulmonary metastasis in cases of tumors infiltrating the intima of the IVC.

## Conclusions

Resected cases of PDAC directly invading the IVC are rare. PD along with wedge resection of the IVC wall for patients with PDAC directly invading the adventitia of the IVC can be performed safely. Further accumulation of cases is needed to elucidate the prognostic impact of IVC invasion.

## Abbreviations

CA19-9: Carbohydrate antigen 19–9
CT: Computed tomography
IVC: Inferior vena cava
PD: Pancreatoduodenectomy
PDAC: Pancreatic ductal adenocarcinoma
POD: Postoperative day
PV: Portal vein
